# Encourage or Reject Employee Involvement: Value Creation in Human Resource Management in the AI Era—An Evolutionary Game Analysis of Enterprises and Employees

**DOI:** 10.3390/bs14121220

**Published:** 2024-12-18

**Authors:** Xiaowei Yang, Shumin Yan

**Affiliations:** School of Economics and Management, Tongji University, Shanghai 200092, China

**Keywords:** artificial intelligence, value co-creation, human resource management, evolutionary game, employee involvement

## Abstract

In the context of globalization and rapid technological advancement, the introduction of Artificial Intelligence (AI) has brought new opportunities and challenges to Human Resource Management (HRM). This study constructs an evolutionary game model to explore the strategy choices and evolutionary paths of enterprises and employees in HRM value co-creation with AI involvement. We numerically simulated the dynamic evolution of strategies under different scenarios, revealing the equilibrium characteristics of strategic interactions between enterprises and employees in the AI context. The study finds that, first, the evolutionary game system between enterprises and employees converges to two equilibrium points: {cooperation, active} and {non-cooperation, passive}. Overall, the probability of the former is 2.39 times greater than that of the latter. Second, higher initial probabilities of cooperation and active involvement, along with lower costs for cooperation and active involvement, facilitate the system’s evolution towards the {cooperation, active} equilibrium. Third, enterprises are more sensitive to the benefit distribution ratio than employees. This study provides theoretical support for effectively conducting HRM practices in the AI era through systematic analysis of HRM value co-creation behavior, along with practical policy recommendations.

## 1. Introduction

In the context of globalization and rapid technological advancement, Human Resource Management (HRM) is undergoing profound transformation. The relationship between companies and employees is shifting from a traditional hierarchical employment model to a more collaborative and co-creative approach, where both enterprises and employees actively participate in decision-making and value creation [1]. The concepts of strategic HRM and value co-creation are increasingly applied in HRM, emphasizing the importance of employee involvement and advocating for value creation between companies and employees [2,3]. Employee involvement in HRM can lead to increased job satisfaction and retention rates, as well as improved organizational performance, but may also bring about challenges such as higher time and financial costs, resource consumption, and conflicts between employees and employers due to misaligned goals [3,4]. Meanwhile, the introduction of Artificial Intelligence (AI) has brought new opportunities and challenges to HRM [5]. AI’s advanced analytics and automation capabilities have significantly altered the interaction between companies and employees [6], profoundly impacting the effectiveness, accuracy, and personalization of HRM practices.

However, existing research primarily focuses on how companies utilize AI to optimize HRM processes, such as recruitment, performance evaluation, and employee training [7], with insufficient exploration of the dynamic mechanisms of value co-creation between companies and employees in the AI environment. Specifically, studies on the strategic choices and evolutionary processes of companies and employees regarding their involvement in HRM activities before and after AI integration are still lacking. This results in a gap in theoretical guidance for effectively leveraging AI to encourage employee involvement and jointly enhance HRM value in practice. While this study primarily focuses on AI-driven HRM, the model proposed here is not limited to AI adoption. It can also be applied to a wider range of technological adoptions, where new technologies—such as automation, digitalization, or other advanced technologies—lead to both increased costs and potential returns for enterprises and employees.

Evolutionary game theory provides an effective tool for analyzing such dynamic interactions [8]. It helps in understanding the costs and benefits of strategic choices made by companies and employees and reveals how strategies evolve over time under different external conditions and incentive mechanisms, in many cases ultimately reaching a stable equilibrium. Based on this, the present study aims to construct an evolutionary game model to explore the strategic choices and evolutionary paths of companies and employees in HRM value co-creation after the introduction of AI. Through quantitative analysis of the model, we identify how AI influences collaborative behaviors between companies and employees, revealing which factors effectively promote or hinder the realization of HRM value co-creation.

Furthermore, the study focuses on modeling the evolutionary game between enterprises and employees to explore the strategic choices and interactions that drive value co-creation in HRM under AI conditions. The granularity of the model is based on a strategic interaction between two main actors: enterprises and employees, where enterprises can choose either a cooperative strategy (involving employees in HRM) or a non-cooperative strategy (independently conducting HRM). Similarly, employees can choose between active participation or passive involvement in HRM activities. These strategies are influenced by the payoffs and costs associated with each strategy, with probabilities determining the likelihood of selecting each strategy. The update rule follows a replicator dynamics model, where the strategies evolve over time based on the fitness (payoffs) of each strategy in the ongoing interaction. Incentive mechanisms, such as feedback from employees and alignment of enterprise goals with employee needs, help drive cooperation and co-creation. The model simulates how these strategic decisions evolve over time to reflect real-world decision-making dynamics under AI-driven HRM systems.

Our research aims to investigate the presence of equilibrium points in the HRM value co-creation game between enterprises and employees in the context of AI, and to explore which equilibrium the system tends towards. Specifically, we seek to identify the key factors that influence the strategic choices of both enterprises and employees and understand how these factors affect the evolution of their strategies. The key findings reveal that the evolutionary game model indicates a convergence towards two equilibrium points: {cooperation, active} and {non-cooperation, passive}, with the former being 2.39 times more likely than the latter. Additionally, higher initial probabilities of cooperation and active participation, along with lower participation and AI adoption costs, are essential for guiding the system towards the cooperative equilibrium. Furthermore, the study shows that enterprises are more sensitive than employees to the distribution of benefits. The results also highlight the critical role of employee participation in achieving higher performance through strategic alignment, providing practical recommendations for managers and employees to design and implement HRM strategies that foster collaboration in an AI-driven environment.

## 2. Literature Review

The concept of value co-creation originally emerged from the service-dominant logic, emphasizing the collaborative creation of value between businesses and customers [9]. In the field of Human Resource Management (HRM), value co-creation refers to the joint involvement of companies and employees in HRM practices to enhance organizational performance and employee satisfaction [10,11]. Specifically, value co-creation manifests in involved decision-making [12], skills development [13] and cultural building [14]. Employee involvement in HRM brings substantial benefits to value co-creation. For example, employees’ active involvement contributes to higher work efficiency and innovation [15], enhances employees’ sense of belonging and satisfaction [16], and reduces turnover rates [17], and diverse perspectives aid in innovative thinking and problem-solving [18]. Employee involvement also ensures the sustainability of HR practices by integrating employees’ feedback, thereby driving innovation and adaptability [19]. However, excessive employee involvement may have negative effects. It can increase time and financial costs [4], consuming significant resources [3]. During the involvement process, conflicting goals between employees and companies can lead to tension [20] and an overload of opinions may complicate decision-making and reduce efficiency. Some studies argue that the effects of employee involvement in HRM depend on specific practices and contexts [21]. They can be enhanced when combined with employee well-being and performance but might face challenges due to differing objectives among management, employees, and regulators.

The application of AI in HRM is becoming increasingly prevalent [22]. AI technology has broad prospects in areas such as recruitment screening, training and development, performance evaluation, and employee-relations management [7]. It presents opportunities for simplifying processes, improving decision-making efficiency, and fostering value co-creation, such as through screening resumes to improve hiring efficiency [23], tailoring training programs to individual employee needs, and assessing performance using big data and machine learning [24]. The application of AI technology in HRM primarily focuses on utilizing machine learning, data analytics, and automation systems to optimize recruitment, employee training, performance evaluation, and incentive mechanisms. Through intelligent decision support, AI helps enterprises achieve personalized management, increase employee engagement, enhance collaboration efficiency, and ultimately promote the joint development of both enterprises and employees [25]. However, the application of AI also brings challenges related to privacy, security, and ethical considerations [26]. Concerns such as potential job displacement and data privacy have been raised in response to the increased use of AI in HRM [27,28].

The importance of studying employee involvement in HRM for value co-creation becomes more pronounced against the backdrop of AI’s widespread application. The main reasons are the following: (1) Human-AI Collaboration: with AI handling large datasets and automating routine tasks, human employees working alongside AI can maximize strengths and improve overall performance [29,30]. (2) Ethical Oversight: AI can assist in decision-making, but human oversight is crucial for interpreting AI outcomes, and employee involvement ensures ethical issues are monitored and addressed [31]. (3) Enhanced AI Adaptation: employee involvement helps increase acceptance of technological changes and adaptability to AI-driven shifts [30]. Thus, studying employees’ behavioral choices regarding HRM value co-creation with AI involvement is of significant theoretical and practical importance.

Evolutionary Game Theory, which combines elements of game theory and evolutionary biology, is a suitable tool for studying dynamic changes in strategy distribution within groups [32]. In management studies, it has been used to analyze the evolution of strategies in repeated interactions between individuals or organizations [33]. In HRM, evolutionary game theory has mainly been applied to labor relations, organizational behavior, and knowledge-sharing dynamics [34]. Therefore, evolutionary game theory is particularly apt for investigating the evolution of HRM value co-creation involving companies and employees with AI. Through evolutionary game models, this study explores how AI affects companies’ and employees’ strategy choices, and benefits distribution, and, ultimately, the equilibrium state of the game. Such insights help understand the profound impacts of AI on HRM and provide theoretical support for better corporate utilization of AI in practice. Nonetheless, current studies have rarely applied evolutionary game theory to analyze value co-creation interactions between companies and employees, particularly with respect to the influence of technological factors like AI on strategy selection.

In summary, the study of HRM value co-creation between companies and employees holds significant theoretical and practical relevance, yet there is a lack of in-depth research on the dynamic mechanisms of strategy interactions. Evolutionary game theory provides an analytical tool for studying these dynamic processes. Considering the influence of AI on companies’ and employees’ behavior in HRM value creation, this study aims to analyze strategy interactions between companies and employees in AI-enabled HRM cooperation, using evolutionary game theory to examine their impact on value co-creation. Next, we will define relevant terms and concepts related to HRM value co-creation and AI in HRM. Following that, we will construct an evolutionary game model considering AI involvement and analyze the game processes under different scenarios through numerical simulations. This will provide theoretical guidance for companies conducting HRM in an AI context, and will contribute to research on the application of evolutionary game theory in HRM.

## 3. Analysis of HRM Value Co-Creation Game Behavior Between Companies and Employees

### 3.1. Definition of Relevant Concepts

HRM value refers to the comprehensive benefits jointly achieved by companies and employees through effective HRM practices, encompassing both enhanced corporate performance and employee personal development [35]. For companies, HRM value is reflected in improved organizational performance, competitive advantage, enhanced innovation capabilities, and the achievement of strategic goals [36]. For employees, HRM value includes increased job satisfaction, more career development opportunities, skill and competency enhancement, and personal fulfillment [37]. Such win–win value creation contributes to building a positive organizational culture and enhancing employee belonging and loyalty.

Employee involvement in HRM value co-creation refers to the active involvement of employees in HRM practices, collaborating with companies to create value [38]. This involvement includes expressing opinions during decision-making, involving oneself in training and development programs, contributing to organizational culture building, and actively providing feedback on HRM practices. Employees’ active involvement not only satisfies their growth needs, but also promotes organizational innovation and adaptability, leading to joint development for both the enterprise and employees [39].

### 3.2. Analysis of Enterprise and Employee Behavior

In the process of HRM value co-creation, enterprises and employees are the two sides of the coin, and their strategic choices directly affect the outcomes of value co-creation and organizational performance.

#### 3.2.1. Enterprises’ Strategy Choices

- Encouraging Employee Involvement: enterprises can choose to encourage employees to actively become involved in HRM practices by establishing open communication channels, providing training and development opportunities, and implementing decision-making mechanisms to promote employee involvement. This strategy helps improve employee satisfaction and a sense of belonging, enhances organizational innovation capabilities and adaptability, and potentially achieves results leading to a mutually beneficial outcome for both enterprises and employees [40]. However, this approach may also increase management costs and extend decision-making times, requiring enterprises to balance resource investment against potential returns.
- Restricting Employee Involvement: enterprises may also limit employee involvement in HRM practices for reasons such as control, efficiency, or confidentiality considerations [41]. This strategy may reduce management costs and simplify processes in the short term, but can lead to a decline in employee motivation, increased turnover rates, decreased innovation capability, and ultimately harm long-term organizational performance [42].

#### 3.2.2. Employees’ Strategy Choices

- Active Involvement: employees actively become involved in HRM practices, including taking part in training, providing suggestions, and involving themselves in decision-making processes, thereby enhancing their skills and career development opportunities [39]. Active involvement helps increase job satisfaction, improve performance, and strengthen organizational identification. However, it may also increase workloads and responsibilities, requiring employees to invest more time and energy [38].
- Passive Attitude: employees may choose not to become involved or passively accept HRM practices, possibly due to a lack of trust, a lack of involvement channels, or dissatisfaction with the enterprise [43]. Although this may temporarily reduce workload, in the long run it could lead to missed career development opportunities, lower job satisfaction, and decreased performance, ultimately affecting both individual and organizational development [37].

## 4. AI-Involved Game Model Between Enterprises and Employees

### 4.1. Research Design

This study employs evolutionary game theory, a framework commonly used to model and analyze strategic interactions where the outcomes of participants’ decisions depend on the choices of others [44]. It focuses on how enterprises and employees, as strategic players, make decisions in the context of HRM value co-creation, particularly with the involvement of AI. The goal is to identify equilibrium points in the system and analyze how factors such as cooperation costs, involvement costs, and the distribution of co-created values influence strategy choices. The research design includes model construction, experimental design, and data collection and processing.

#### 4.1.1. Model Construction

The study develops an evolutionary game model that incorporates AI as a key factor influencing HRM value co-creation. The model includes two main players—enterprises and employees—each with strategic decisions based on the benefits and costs of cooperation. Key parameters considered in the model are the initial strategy probabilities, cooperation costs, involvement costs, AI profit distribution ratios, and co-created value distribution ratios. The evolutionary process of these strategies is simulated to understand the dynamic shifts between equilibrium points such as {cooperation, active} and {non-cooperation, passive}.

#### 4.1.2. Experimental Design

For the experimental design, the study uses numerical simulations with MATLAB R2023a to explore the evolution of strategies under different conditions. Various scenarios are simulated by adjusting parameters like the initial probabilities of cooperation and active involvement, cooperation costs, and the distribution of AI-related benefits. The aim is to understand how these parameters influence the strategic choices of both enterprises and employees, particularly in guiding the system towards the desired equilibrium of cooperation and active involvement.

#### 4.1.3. Data Sources

The data used in this study are primarily simulation-based, not empirical. The initial model parameters were determined through the Delphi method, which involved input from experts in human resource management, enterprise executives, and middle management personnel. The experts assessed the relative importance of different factors, and the results were averaged from 12 independent evaluations. Simulation data were then used to test how different parameter configurations influence the evolutionary process, helping to validate the model’s predictions.

#### 4.1.4. Data Processing

During data processing, special attention was given to the accuracy and consistency of the simulation data. Multiple runs of the simulation model in MATLAB ensured the stability and reproducibility of the results. All data were standardized, and sensitivity analysis was conducted on key parameters to ensure the model’s adaptability to various scenarios.

### 4.2. Assumptions of the AI-Involved Game Model Between Enterprises and Employees

This paper assumes that both enterprises and employees exhibit bounded rationality in the game, meaning that their strategic choices have a certain level of randomness. Through repeated interactions in the game, they gradually approach a stable state over the long term through adaptation and revenue optimization processes. Both enterprises and employees act according to their behavior logic, to maximize their respective payoffs. The model also assumes that these behavioral choices occur within a specific environmental and institutional context. Other assumptions are as follows:

1. Assumption 1. The strategies of enterprises can be divided into independently conducting HRM (non-cooperation): the enterprise independently formulates and implements HRM strategies without considering employee involvement. This may result in strategies that do not align with employee needs, thereby affecting performance. Alternatively, enterprises can choose to involve employees in HRM (cooperation): enterprises and employees jointly formulate and implement HRM strategies, promoting value co-creation and improving performance, denoted as {non-cooperation, cooperation}. Employee strategies are divided into active involvement (active): employees actively become involved in HRM activities, provide constructive feedback, and enhance work efficiency and innovation capabilities. Employees can also choose passive involvement (passive): they may resist, be inefficient, or not become involved in HRM activities, denoted as {active, passive}. Given the evolution of enterprise HRM practices, with the spread of scientific management and the integration of AI in HRM, management strategies generally tend to shift “from non-cooperation to cooperation”. To reflect this feature, we denote the probability of enterprises choosing a cooperative strategy as p and a non-cooperative strategy as 1 − p, where p and (1 − p) reflect the tendency of enterprises to adjust their strategies over time, based on payoffs. Similarly, the probability of employees choosing an active strategy is denoted by q, while a passive strategy is 1 − q, where 0 ≤ p, q ≤ 1. It should be noted that these probabilities reflect the tendency of enterprises and employees to adjust their strategies based on their payoffs over multiple interactions, influenced by cooperation costs and the behavior of their counterparts.
2. Assumption 2. Before AI became involved in HRM value creation, enterprises managed HRM independently, saving resources that would have been used to promote employee involvement. This resulted in the rapid implementation of strategies in the short term, yielding a return of $R_{1}$. However, due to the lack of employee feedback and involvement, the effectiveness of strategies might be compromised, leading to increased hidden costs, and the cost of HRM management is denoted by $C_{1}$. When enterprises and employees collaborate on HRM value creation, the active involvement of employees makes HRM strategies more effective, enhancing organizational performance, with the return being $R_{2}$ At the same time, enterprises need to invest more resources (such as time and training) to promote employee involvement, resulting in an HRM management cost of $C_{2}$. Typically, $R_{1}$ < $R_{2}$, and $C_{1}$ < $C_{2}$. When employees choose not to become involved in enterprise HRM management and merely passively accept HRM policies, they save time and energy, resulting in a cost of $c_{1}$. However, they miss out on career development opportunities, which impacts long-term career growth, resulting in a relatively lower return of $r_{1}$. On the other hand, when employees choose to actively become involved in enterprise HRM management, they need to invest extra time and energy into HRM activities, with a cost of $c_{2}$. However, they gain career development opportunities and increased satisfaction, leading to a return of $r_{2}.$ Typically, $c_{1}$ < $c_{2}$, and $r_{1}$ < $r_{2}.$ (Note that uppercase variables represent enterprises, while lowercase variables represent employees.)
3. Assumption 3. After AI’s involvement in HRM value creation, its impact on enterprises manifests as increased returns. AI technology can improve HRM efficiency and accuracy, optimizing talent management, with returns increasing to $R_{2}$′. Specifically, $R_{2}$ < $R_{2}$′. Introducing AI entails upfront investments in hardware, software, and employee training, which result in increased initial costs, denoted by $C_{2}$′, $C_{2}$ < $C_{2}$′. Therefore, $R_{2}$′ = $R_{2}$ + Δ$R_{AI}$, and $C_{2}$′ = $C_{2}$ + Δ$C_{AI}$, where Δ$R_{AI}$ represents the increase in enterprise returns brought about by AI, and Δ$C_{AI}$ represents the rise in costs incurred by the introduction of AI, with Δ$R_{AI}$, Δ$C_{AI}$ < 0.
4. Regarding the impact of AI on employees: when employees actively become involved in HRM management, AI assistance makes their involvement more efficient, providing personalized career development support, which increases returns to $r_{2}$′, where $r_{2}$ < $r_{2}$′. AI simplifies involvement processes, reducing the required time and energy investment, thereby decreasing costs to $c_{2}$′, where $c_{2}$′ < $c_{2}$. Here, $r_{2}$′ = $r_{2}$ + Δ$r_{AI}$, and $c_{2}$′ = $c_{2}$ − Δ$c_{AI}$, where Δ$r_{AI}$ represents the increase in returns brought by AI, and Δ$c_{AI}$ represents the decrease in costs brought by AI, with Δ$r_{AI}$, Δ$c_{AI}$ > 0.
5. When employees passively become involved in HRM management, due to enterprise monitoring and AI surveillance, negative behaviors are more easily identified, and their short-term returns might decrease to $r_{1}$′, where $r_{1}$′ < $r_{1}$. Passive behavior may also bring greater career risks, increasing costs to $c_{1}$′, where $c_{1}$ < $c_{1}$′.
6. Assumption 4. The returns generated after AI involvement, ΔAI, are distributed proportionally, with a proportion of a for enterprises and 1−a for employees, where 0 ≤ a ≤ 1. After enterprises and employees collaborate in HRM management, there will be a co-created value, denoted by Δ$R_{h}$, which is also allocated proportionally, with the enterprise receiving a proportion of b and employees receiving 1 − b, where 0 ≤ b ≤ 1. When enterprises choose collaboration while employees opt for passive involvement, enterprises can still benefit from the automation and efficiency improvements brought by AI, gaining k ΔAI, where 0 < k < 1. When enterprises choose not to collaborate, but employees choose active involvement in HRM practices, even though comprehensive support from the enterprise may be lacking, this effort may still positively affect enterprise performance, such as by improving work efficiency and reducing internal friction. Therefore, the enterprise may receive an additional incremental benefit, ΔR, which, although small, reflects the positive impact of employees’ proactive attitudes on enterprise performance;
7. Assumption 5. The returns from collaboration are greater than those from non-collaboration, but the costs associated with collaboration are also higher. For enterprises, $R_{2}$ − $C_{2}$ > $R_{1}$ − $C_{1}$. For employees, engaging actively in HRM activities requires employees to invest more time and effort, which might result in reduced net gains in the short term. However, in the long term, active involvement is beneficial for career development and personal growth. These long-term gains are considered non-direct benefits in this model, and are not reflected in the calculation of short-term net gains, where $r_{2}$ − $c_{2}$ ≤ $r_{1}$ − $c_{1}$.
8. Assumption 6. After the introduction of AI, the marginal returns from collaboration increase, and costs may be reduced. For enterprises, $R_{2}$′ − $C_{2}$′ > $R_{2}$ − $C_{2}$. For employees, $r_{2}$′ − $c_{2}$′ > $r_{2}$ − $c_{2}$.
9. Assumption 7. AI technology makes information more symmetric between enterprises and employees, reducing barriers to collaboration. This increased symmetry leads to more efficient communication and trust-building, facilitating cooperation. While this assumption does not require direct mathematical encoding, its influence is reflected in the overall tendency for enterprises and employees to collaborate more effectively due to reduced communication costs and improved mutual understanding.

### 4.3. Strategy Choices of Enterprises and Employees with AI Involvement

Based on the method proposed by Friedman [45], the Jacobian matrix, derived from the replicator dynamics equations, and the local stability of the payoff matrix can be used to verify whether the strategy combinations formed by both sides of the game are Evolutionarily Stable Strategies (ESSs), and to analyze which factors will influence the strategy choices of both parties. Under our model of AI involvement, the payoff matrix for enterprises and employees is shown in Table 1.

Assuming that the expected payoffs for enterprises adopting the “non-cooperation” and “cooperation” strategies are $E_{1}$ and $E_{2}$, respectively, and the expected payoffs for employees adopting the “non-involvement” and “active involvement” strategies are $e_{1}$ and $e_{2}$:


(1) $$E_{1}=q\;(R_{1}+\Delta R-C_{1})+(1-q)\;(R_{1}-C_{1})$$

(2) $$E_{2}=q\;[R_{2}+a\Delta \mathrm{AI}+b\Delta R_{h}-(C_{2}+\Delta C_{AI})]+(1-q)\;[R_{2}+k\;\Delta \mathrm{AI}-(C_{2}+\Delta C_{AI})]$$

(3) $$e_{1}=p\;(r_{1}\prime -c_{1}\prime )+(1-p)(r_{1}-c_{1})$$

(4) $$e_{2}=p\;[r_{2}+(1-a)\;\Delta \mathrm{AI}+(1-b)\;\Delta R_{h}-(c_{2}-\Delta c_{AI})]+(1-p)\;(r_{2}-c_{2})$$

Thus, under our model of AI involvement, the replicator dynamic equation for enterprises adopting the “cooperation” strategy is derived from the evolutionary game framework, where the strategy evolution depends on the relative payoffs of the different strategies:


(5) $$F(p)\;=\frac{d_{p}}{d_{t}}=\;p\;(E_{2}-E_{avg})$$

F(p): The rate of change in the proportion of enterprises choosing the ‘cooperation’ strategy over time reflects the replicator dynamic for enterprises.

$E_{2}$: The expected payoff for enterprises choosing the “cooperation” strategy.

$E_{avg}$: The overall expected payoff for enterprises, calculated as a weighted average of the expected payoffs for choosing “cooperation” and “non-cooperation” strategies.

$E_{2}$ − $E_{avg}$: The fitness difference between cooperative and non-cooperative strategies,

(6) $$E_{avg}=\;pE_{2}+(1-p)E_{1}$$

The replicator dynamic equation for employees choosing the “cooperation” strategy is

(7) $$F(q)=\;\frac{d_{q}}{d_{t}}\;=q\;(e_{2}-e_{avg})$$

F(q): reflects the replicator dynamic for employees.

$e_{2}$: the expected payoff for employees choosing the “active involvement” strategy.

$e_{avg}$: the overall expected payoff for employees, calculated as a weighted average of the expected payoffs for choosing the “active involvement” and “passive” strategies.

$e_{2}$ − $e_{avg}$: the fitness difference between active and passive strategies:

(8) $$e_{avg}=qe_{2}+(1-q)e_{1}$$

To determine the equilibrium points of the replicator dynamic equations, we need to identify the stable points that the system reaches in the long term, i.e., where the rate of change for all replicator dynamic equations is zero. These equilibrium points indicate that the proportion of strategies within the population no longer changes, thus reaching a stable state.

To find the equilibrium points, we set the replicator dynamic equations for enterprises adopting the “cooperation” strategy and employees choosing the “active involvement” strategy both equal to zero. We then analyze the solutions to these equations to determine the potential equilibrium points.

The replicator dynamic equation for enterprises adopting the “cooperation” strategy is F(p) = p(1 − p) ($E_{2}$ − $E_{avg}$) = 0, and it has three possible solutions:

p = 0: this indicates that enterprises do not choose the “cooperation” strategy at all, meaning that all enterprises choose “non-cooperation”.

p = 1: this indicates that enterprises fully adopt the “cooperation” strategy, meaning that all enterprises choose “cooperation”.

$E_{2}$ = $E_{avg}$: this means that the expected payoffs from adopting the “cooperation” and “non-cooperation” strategies are equal, implying that no matter which strategy is chosen, the payoff is the same. Therefore, the population maintains a mixed strategy equilibrium.

The replicator dynamic equation for employees adopting the “active involvement” strategy is F(q) = q(1 − q) ($e_{2}$ − $e_{avg}$) = 0, and it has three possible solutions:

q = 0: this indicates that employees do not choose the “active involvement” strategy at all, meaning that all employees choose “passive involvement”.

q = 1: this indicates that employees fully adopt the “active involvement” strategy, meaning that all employees choose “active involvement”.

$e_{2}$ = $e_{avg}$: this means that the expected payoffs from adopting the “active involvement” and “passive involvement” strategies are equal, implying that no matter which strategy is chosen, the payoff is the same. Therefore, the population maintains a mixed strategy equilibrium.

Through the solutions of the replicator dynamic equations, we can identify the following five equilibrium points in the game:

Global Equilibrium Points:

**A**(0, 0): the enterprise chooses “Non-cooperation”, and employees choose “Passive Involvement”.

**B**(0, 1): the enterprise chooses “Non-cooperation”, and employees choose “Active Involvement”.

**C**(1, 0): the enterprise chooses “Cooperation”, and employees choose “Passive Involvement”.

**D**(1, 1): the enterprise chooses “Cooperation”, and employees choose “Active Involvement”.

Mixed Strategy Equilibrium Point:

**E**($x_{0}$, $y_{0}$): where $x_{0}$ ∈ (0, 1) and $y_{0}$ ∈ (0, 1), indicating that the enterprise and employees adopt a mixed proportion between cooperation and non-cooperation, and between active and passive involvement.

Solving for the internal equilibrium point **E** ($x_{0}$, $y_{0}$), we obtain the coordinates of the internal equilibrium point **E** ($x_{0}$, $y_{0}$) as follows:

(9) $$x_{0}=p_{0}=-\frac{\left(r_{2}-c_{2})-(r_{1}-c_{1}\right)}{\left[\Delta c_{AI}+(1-a)\Delta AI+(1-b)\Delta R_{h}]-(\Delta r_{1}-\Delta c_{1}\right)}$$

(10) $$y_{0}\;=q_{0}=-\frac{(R_{2}-C_{2}-\Delta c_{AI})-(R_{1}-C_{1})+k\Delta AI}{a\Delta AI+b\Delta R_{h}-k\Delta AI-\Delta R}$$

### 4.4. Stability Analysis of Equilibrium Points in the Game Between Enterprises and Employees Under AI Involvement

To analyze the stability of the equilibrium points, we need to construct the Jacobian matrix and evaluate the eigenvalues of each equilibrium point. The sign (positive or negative) of the eigenvalues will determine the stability of each equilibrium point. The two state variables of the replicator dynamic system are p and q, representing the proportion of enterprises choosing “cooperation” and the proportion of employees choosing “active involvement”, respectively. The corresponding replicator dynamic equations are


(11) $$F(p)=\frac{d_{p}}{d_{t}}=p(1-p)\;(E_{2}-E_{1})=p(1-p)\;D(p)$$

(12) $$F(q)=\;\frac{d_{q}}{d_{t}}\;=q(1-q)\;(e_{2}-e_{1})=q(1-q)\;d(q)$$

(13) $$D(p)=E_{2}-E_{1}=D_{1}+qD_{2}$$

(14) $$d(q)=e_{2}-e_{1}=d_{1}+{\mathrm{pd}}_{2}$$

$In\;this\;model,\;we\;define\;D_{1}\;and\;D_{2}$ to represent parameters related to enterprises,

(15) $$D_{1}=(R_{2}-C_{2}-\Delta c_{AI})-(R_{1}-C_{1})+k\Delta AI$$

(16) $$D_{2}=a\Delta AI+b\Delta R_{h}-k\Delta AI-\Delta R$$

while $d_{1}\;and\;d_{2}$ represent parameters related to employees.


(17) $$d_{1}=\left(r_{2}-c_{2}\right)-\left(r_{1}-c_{1}\right)$$

(18) $$d_{2}=\Delta c_{AI}+(1-a)\Delta AI+(1-b)\Delta R_{h}-(\Delta r_{1}-\Delta c_{1})$$

The Jacobian matrix is composed of elements that are partial derivatives of the replicator dynamic equations, with respect to the state variables:

(19) $$J=\left[\begin{array}{cc} \frac{\partial F(p)}{\partial p} & \frac{\partial F(p)}{\partial q}\\ \frac{\partial F(q)}{\partial p} & \frac{\partial F(q)}{\partial q} \end{array}\right]$$

After computing $\frac{\partial F(p)}{\partial p}$, $\frac{\partial F(p)}{\partial q}$, $\frac{\partial F(q)}{\partial p}$, $\;and\;\frac{\partial F(q)}{\partial q}$, the Jacobian matrix is

(20) $$J=\left[\begin{array}{cc} \left(1-2p)\;D(p\right) & p\;(1-p)D_{2}\\ q(1-q)d_{2} & \left(1-2q)\;d(q\right) \end{array}\right]$$

The determinant and trace expressions of the Jacobian matrix are calculated for each equilibrium point to conduct stability analysis, resulting in the numerical expressions and stability analysis of the five equilibrium points, as shown in Table 2 and Table 3. The relevant parameters are presented in Table 4. When assigning values to model parameters, in order to obtain data closer to management practice in theory and practice, and to avoid the aura of authoritative experts in collective discussion and prevent bias, we adopt the Delphi method to collect expert opinions and enhance the reliability of parameter setting process [46]. The initial parameter settings are determined using the expert scoring method, involving evaluations by university professors specialized in HRM, enterprise executives, and middle management personnel. The data received from 12 evaluations are averaged to be used as the initial parameter values.

**Figure 1:**
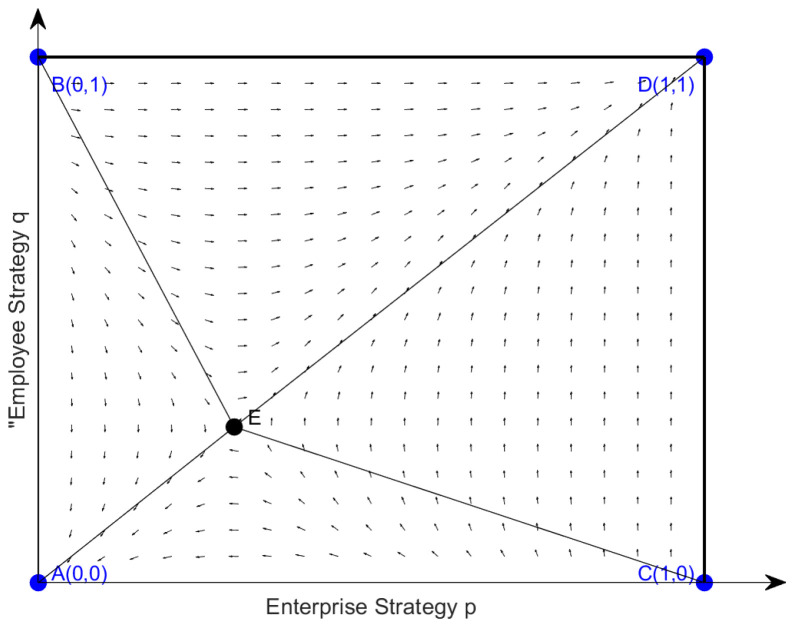
Evolutionary phase diagram of enterprises and employees.

The evolutionary equilibrium results indicate that equilibrium points **A**(0, 0) and **D**(1, 1) are both ESS equilibrium points, representing a situation where the enterprise chooses “Non-cooperation” and the employees choose “Non-involvement”. If the initial state of the system is close to **A**(0, 0), where both enterprises and employees choose not to cooperate or involve, the system will converge to this state. Conversely, if the initial state is close to **D**(1, 1), where both parties choose cooperation and active involvement, the system will also converge to this state. Equilibrium points **C**(0, 1) and **D**(1, 0) are both unstable, meaning the system will not remain at these states in the long term. The internal equilibrium point **E**($p_{0}$, $q_{0}$) is a saddle point, playing a key role in determining whether the system evolves towards equilibrium points **A**(0, 0) or **D**(1, 1). While **E**($p_{0}$, $q_{0}$) is unstable, it serves as a critical decision point: the system moves away from E and converges to either **A** or **D**, depending on initial conditions and parameter settings. This reflects how small changes in cooperation costs, incentive structures, and employee involvement can influence system outcomes in real-world AI-driven HRM scenarios. Unlike traditional models like the Prisoner’s Dilemma, which are based on static payoffs, our model incorporates dynamic factors, such as AI-enhanced cooperation incentives, making the system more adaptable and realistic. Figure 1 illustrates the system’s trajectory, showing how the unstable equilibrium leads the system towards one of the stable outcomes.

As shown in Figure 1, in the illustrated phase diagram, the horizontal axis represents the enterprise strategy p, and the vertical axis represents the employee strategy q, both ranging from [0,1]. The arrows in the diagram indicate the direction of system dynamics, showing the evolutionary trends of the strategy combinations between enterprises and employees. Key parameters $D_{1}$ = −8, $D_{2}$ = 27, $d_{1}$ = −15, and $d_{2}$ = 51 play an important role in strategy evolution. The internal equilibrium point **E**($p_{0}$, $q_{0}$) is located at $p_{0}$≈ 0.2941, $q_{0}$ ≈ 0.2963, and the black solid lines connecting point **E** with points **A**, **B**, **C**, **D** divide different strategy regions. Since $D_{2}$ and $d_{2}$ are positive and relatively large, it indicates that the benefits of enterprises and employees are highly dependent on the cooperation level of the other side. The negative values of $D_{1}$ and $d_{1}$ indicate that unilateral cooperation may lead to losses if the other side does not cooperate.

In summary, the strategy choices of enterprises and employees tend to converge on either mutual cooperation or mutual non-cooperation. The relative attractiveness of cooperation versus non-cooperation is influenced by factors such as the costs and benefits of each strategy (e.g., $R_{2}$ vs. $R_{1}$, $C_{2}$ vs. $C_{1}$ for enterprises, and $r_{2}$ vs. $r_{1}$, $c_{2}$ vs. $c_{1}$ for employees), as well as the shared benefits of collaboration (Δ$R_{h}$) and the costs and benefits associated with AI adoption (ΔAI, Δ$C_{AI}$). Based on the calculated areas, the BECD region (representing mutual cooperation) has an area of 0.7048, while the ABEC region (representing mutual non-cooperation) has an area of 0.2952. This means that the cooperative equilibrium is significantly more attractive, with the area of BECD being approximately 2.39 times larger than the area of ABEC. This suggests that, under the current parameter settings, the system is much more likely to converge towards the cooperative equilibrium. The difference in area sizes highlights how factors such as the AI-related benefits and costs, as well as the distribution of cooperative gains, can influence the system’s trajectory. By adjusting key parameters such as Δ$R_{h}$, ΔAI, and Δ$C_{AI}$, interventions can effectively steer the system towards the desired cooperative outcome. These parameters play a crucial role in shaping the strategy choices of enterprises and employees, and should be further explored in future research to better understand their impact on strategy selection.

## 5. MATLAB Numerical Simulation

To further investigate the interrelationship between parameters that influence the value co-creation of HRM by enterprises and employees, as well as the impact of key factors on evolutionary paths, this study utilizes MATLAB R2023a to simulate the evolutionary paths of strategy choices by enterprises and employees under different parameter settings.

### 5.1. The Effect of Initial Strategy Selection Probabilities on the Evolutionary Path of Opponents in the Game

**Figure 2:**
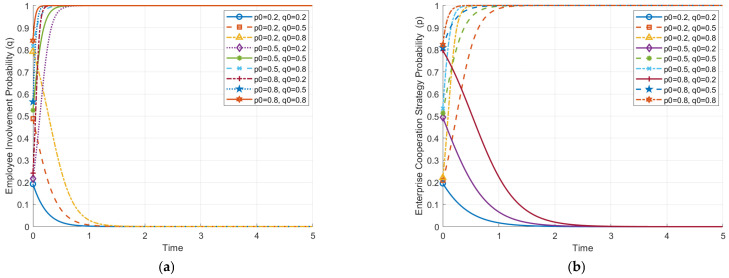
Impact of initial strategy-selection probability on the evolution paths of game opponents. (**a**) Impact of initial enterprise strategy-selection probability on employee-strategy evolution path. (**b**) Impact of initial employee strategy-selection probability on enterprise-strategy evolution path.

Figure 2 illustrates how the initial probabilities of strategy choice by enterprises and employees affect the evolutionary paths of their strategies. These evolutionary paths demonstrate how interactions between enterprises and employees under different initial conditions influence their eventual stable strategic state.

In Figure 2a, the effect of the initial probability of the enterprise choosing cooperation ($p_{0}$) on the evolution path of employee strategies (q) is shown. When the initial probability of cooperation by enterprises is high ($p_{0}$ > 0.5), the evolution of employee strategies shows a trend towards active involvement (q rapidly approaching 1). This indicates that cooperative behavior from enterprises can stimulate employees’ willingness to become involved actively. On the other hand, when the initial cooperation probability of enterprises is low, employees’ probability of active involvement significantly decreases (q drops to 0), suggesting that a non-cooperative stance from enterprises diminishes employees’ motivation.

Figure 2b shows the effect of the initial probability of employees choosing active involvement ($q_{0}$) on the evolution of enterprise strategies (p). It is evident that when the initial probability of active involvement by employees is high ($q_{0}$ > 0.5), enterprise strategies evolve towards cooperation (p rapidly approaching 1), meaning that high levels of employee involvement motivate enterprises to prefer cooperative strategies during the evolution. However, when the initial probability of active involvement by employees is low ($q_{0}$ < 0.5), enterprise strategies gradually evolve towards non-cooperation (p drops to 0), indicating that insufficient employee involvement reduces enterprises’ incentive to cooperate.

Overall, both graphs indicate a significant reciprocal influence between enterprises and employees. The higher the initial probabilities of cooperation or active involvement, the more likely both parties are to evolve towards a cooperative and actively involving stable state, i.e., ESS (1, 1). As shown in Figure 1, when the initial probabilities of cooperation ($p_{0}$) and active involvement ($q_{0}$) are high, corresponding to the upper-right region of the phase diagram (ABCD), the system tends to converge towards the stable cooperation equilibrium **D**(1, 1), where both enterprises and employees adopt cooperative and active involvement strategies. Conversely, if both parties’ initial choices are at lower levels, the system will converge to a non-cooperative, non-involving state, i.e., ESS (0, 0), representing an inefficient equilibrium. This is depicted in Figure 1, where low initial probabilities of cooperation ($p_{0}$) and involvement ($q_{0}$) lead the system towards the lower-left region, corresponding to **A**(0, 0). Therefore, enhancing the initial probability of cooperation and involvement plays a crucial role in achieving a win–win outcome through cooperation. These results emphasize the importance of initial conditions in determining the system’s trajectory. Although the figure does not directly show how adjustments to factors like cooperation benefits or AI adoption costs influence the system, such interventions could impact the likelihood of convergence towards the “cooperative equilibrium”.

### 5.2. Impact of Enterprise Cooperation Cost on Its Evolution Path

**Figure 3:**
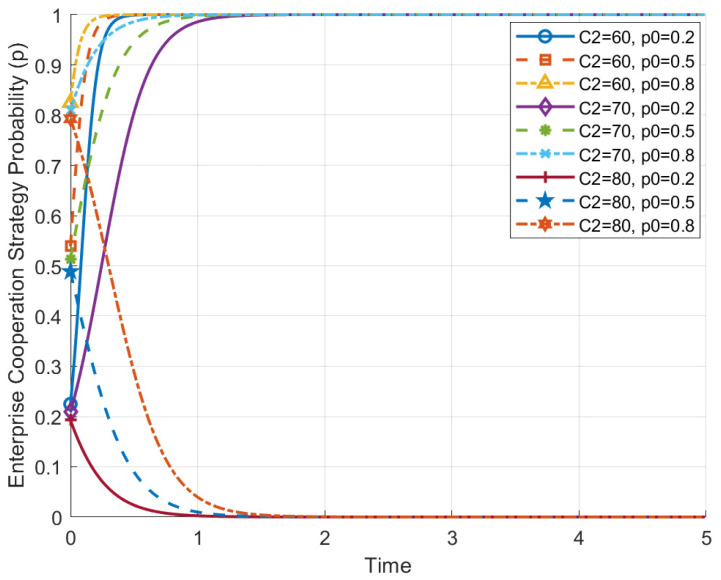
Impact of enterprise cooperation cost on its evolution path.

Figure 3 shows the impact of different enterprise cooperation costs ($C_{2}$ = 60, 70, 80) on the evolution path of the enterprise’s cooperative strategy, based on its initial strategy selection probability ($p_{0}$). The figure illustrates that the level of cooperation cost has a significant effect on the evolution of enterprise cooperation strategies.

When the cooperation cost ($C_{2}$) is relatively low (e.g., $C_{2}$ = 60), the evolution path of the enterprise’s cooperative strategy tends to converge more easily towards cooperation (p approaches 1 quickly). This suggests that low cooperation cost can effectively motivate enterprises to adopt a cooperative strategy to achieve higher benefits. Concrete examples of such costs include expenses related to employee training, communication and coordination efforts, and time spent on negotiating or aligning organizational goals. These costs can be lowered through effective policy and management measures, such as investing in employee development programs, improving communication infrastructure, and streamlining decision-making processes to reduce time and resource allocation. Additionally, incentives such as performance-based rewards for cooperation and the integration of AI to automate routine tasks can further reduce cooperation-related costs.

Conversely, when the cooperation cost is high ($C_{2}$ = 80), the evolution path of the enterprise’s cooperative strategy shows a distinct downward trend, especially when the initial cooperation probability ($p_{0}$) is low (e.g., $p_{0}$ = 0.2 and $p_{0}$ = 0.5). High cooperation cost makes enterprises more inclined to abandon cooperation during evolution, reflecting the inhibitory effect of rising costs on cooperation willingness. Even when the initial cooperation probability is high ($p_{0}$ = 0.8), high cooperation cost still leads to a gradual reduction in the enterprise’s cooperation level, potentially resulting in abandoning cooperation entirely.

Overall, cooperation cost has a significant impact on the evolution path of enterprise strategy. When cooperation cost is low, enterprises are more likely to maintain cooperation and evolve towards a fully cooperative state; whereas, when cooperation cost is high, enterprises tend to abandon cooperation. Thus, reducing cooperation cost (e.g., through technological innovation, improving efficiency, etc.) will facilitate more effective cooperation between enterprises and employees, ultimately promoting the system’s evolution towards a stable equilibrium at (1, 1).

### 5.3. Impact of Employees’ Cost for “Active Involvement” on Their Evolutionary Path

**Figure 4:**
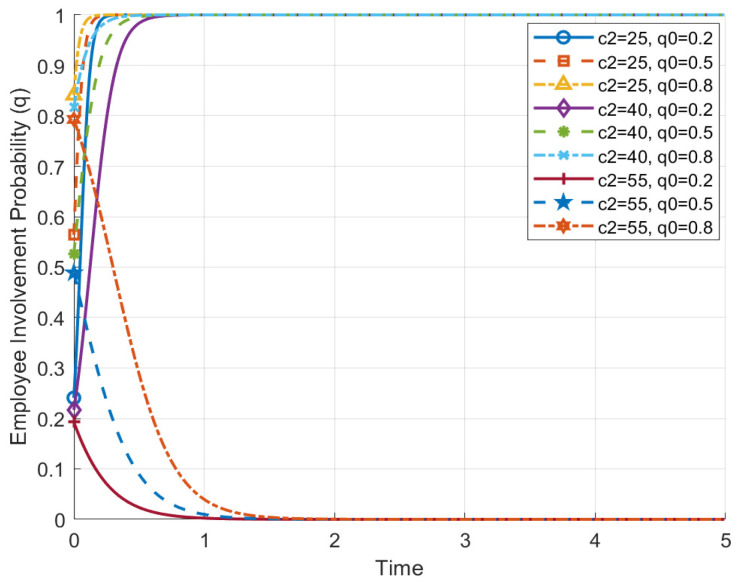
The impact of employees’ active involvement cost on their strategy evolution path.

Based on Figure 4, which shows “the impact of employees’ active involvement cost ($c_{2}$) on their strategy evolution path (q)”, we can observe that the cost of active involvement significantly influences employees’ strategy choices. Specifically, under different cost conditions, the probability of employees’ active involvement evolves over time, displaying various trends. The curves in the figure clearly illustrate the differences in employees’ strategy choices and evolution under low-, medium-, and high-cost conditions, as well as the significant impact of cost on the adoption of active involvement strategies.

When the cost of employees’ active involvement is relatively low ($c_{2}$ = 25), the curves in the figure show that, regardless of the initial active involvement probability ($q_{0}$), employees’ strategies eventually converge to the state of active involvement (q approaches 1). This indicates that when involvement costs are low, employees are more inclined to continue actively involving themselves in HRM activities, as they can obtain greater net benefits from active involvement. However, when the cost of active involvement increases ($c_{2}$ = 40 and $c_{2}$ = 55), the curves tend towards lower involvement probabilities, especially under high-cost conditions ($c_{2}$ = 55), where even if the initial active involvement probability is high ($q_{0}$ = 0.8), the final strategy evolution tends to a lower level, approaching zero. This suggests that high involvement costs can greatly reduce employees’ willingness to actively become involved, leading them to favor passive involvement in the long term. This observation is consistent with the cost–benefit balance principle in game theory: when the cost of involvement is too high, employees choose not to become involved, to avoid incurring more costs.

These results further support the hypothesis in this paper that employees’ strategy choices are influenced not only by the enterprise’s cooperation strategy, but also by their own involvement costs. When enterprises aim to increase employee engagement, reducing the cost of active involvement (e.g., through incentive mechanisms, process simplification, etc.) is an effective approach. By lowering involvement costs, employees can be encouraged to engage more willingly in the enterprise’s HRM activities, thereby achieving a cooperative and mutually beneficial Evolutionarily Stable State (ESS (1, 1)).

### 5.4. Impact of AI Benefit Distribution on the Evolutionary Paths of Enterprises and Employees

**Figure 5:**
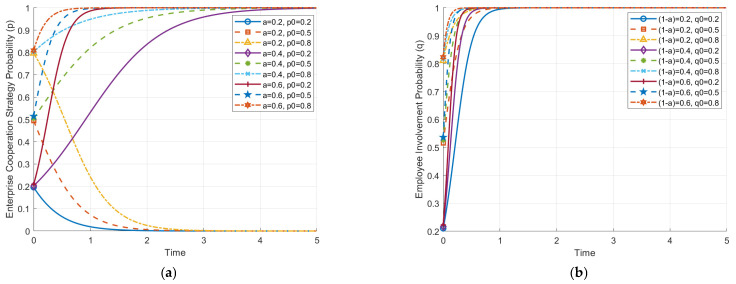
Impact of AI benefit distribution on the evolutionary paths of enterprises and employees. (**a**) Impact of AI benefit distribution on the evolutionary path of enterprises. (**b**) Impact of AI benefit distribution on the evolutionary path of employees.

Based on the analysis of Figure 5, we observe significant differences in the sensitivity of enterprises and employees to the benefit distribution ratio after AI is introduced, each with its own underlying reasons. First, enterprises are more sensitive to the benefit distribution ratio (a) because their cooperation strategy involves relatively high costs (e.g., training, resource allocation), as well as potential management risks. Only when the benefit distribution ratio is high enough can enterprises cover these costs and achieve higher returns, leading to sensitivity to changes in the distribution ratio during evolution. When the benefit distribution to enterprises is low, their willingness to cooperate quickly diminishes, eventually leading to the choice of non-cooperation.

Employees, on the other hand, are relatively less sensitive to the benefit distribution ratio (1 − a). As shown in the figure, regardless of whether the benefit ratio is 0.2, 0.4, or 0.6, the final probability of positive employee involvement converges to 1. This is because the involvement cost for employees is relatively low, and the benefits of positive involvement include not only short-term economic returns, but also long-term gains, such as career development opportunities and skill enhancement. Therefore, as long as there is some benefit, employees are inclined to become involved actively. While this result highlights a general behavior of employees, it is particularly relevant in the context of AI-driven HRM practices. AI can provide more personalized career development pathways, skill-based training, and tailored feedback, which make the long-term benefits of involvement more attractive to employees. This is distinct from traditional HRM technologies, where such personalized benefits might not be as easily achieved.

Overall, enterprises tend to choose non-cooperation if the benefit distribution is insufficient to cover costs, whereas employees, due to the relatively low involvement cost and the more diverse benefits, are willing to become involved actively, even when the benefit ratio is low. The different behaviors of enterprises and employees in strategy selection reflect their respective cost–benefit trade-offs. For example, enterprises may decide against cooperation if the cost of employee training and communication outweighs the expected benefits. In contrast, employees may be willing to invest time and effort in training if they perceive long-term career growth opportunities through AI-enhanced skill development, even if the short-term financial rewards are limited. This provides insight for enterprises in implementing AI technology and motivating employee cooperation—reasonable benefit distribution and incentive mechanisms are key to achieving stable cooperation between both parties.

### 5.5. Impact of HRM Co-Created Value Distribution on the Evolution Pathways of Enterprises and Employees

**Figure 6:**
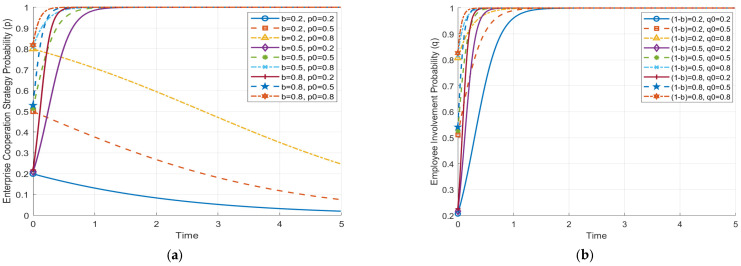
Impact of co-created value distribution on the evolution paths of enterprises and employees. (**a**) Impact of co-created value distribution on enterprise evolution path. (**b**) Impact of co-created value distribution on employee evolution path.

As shown in Figure 6, both the evolution pathways of enterprise strategy and employee strategy exhibit a general trend toward cooperation or positive involvement. This indicates that in the presence of co-created values, both parties are more inclined to cooperate, thereby improving overall performance. The presence of co-created values makes cooperation between enterprises and employees beneficial, and these gains, when appropriately distributed, can effectively incentivize both parties to cooperate and become involved actively.

When the proportion of co-created values received by the enterprise is relatively high (b = 0.8), the probability of the enterprise adopting a cooperative strategy (p) tends to remain high or even approach 1. This is because a higher proportion of co-created values allows the enterprise to obtain more direct benefits from cooperation, thus motivating the enterprise to maintain a cooperative strategy. Conversely, when the enterprise receives a lower share of co-created values (b = 0.2), the probability of cooperation gradually declines over time. This happens even when the initial probability of cooperation is high. This suggests that the enterprise is highly sensitive to cooperative gains, and the distribution proportion significantly affects whether it continues to cooperate.

When the proportion of co-created values received by employees is high (1 − b = 0.8), the probability of employees choosing an active involvement strategy (q) quickly approaches 1 during the evolution process. This implies that a higher distribution ratio encourages employees to actively become involved, thereby promoting collaboration efficiency and quality within the enterprise. Even with a low initial positive involvement probability ($q_{0}$ = 0.2), employees become more inclined to switch to an active involvement state over time. This indicates that a high proportion of co-created values has a significant effect in motivating employee behavior, and employees respond sensitively to the distribution, especially when the proportion is high, substantially enhancing their motivation for active involvement. For instance, in HRM practices, companies that adopt performance-based incentive programs such as profit-sharing or team-based bonuses can motivate employees to contribute more actively to organizational goals. A concrete example is the use of gain-sharing plans in some manufacturing companies, where employees receive a portion of the profits generated from team performance. This approach not only aligns the goals of the company and employees, but also incentivizes employees to work toward the organization’s success.

In summary, a reasonable distribution of co-created values is a key factor in promoting cooperation between enterprises and employees. A higher share of co-created values can incentivize both parties to increase their motivation to cooperate, thereby achieving a win–win situation in terms of enterprise performance and employee satisfaction. This result is consistent with the hypothesis of this study, indicating that the introduction of a reasonable co-created value distribution mechanism in HRM strategies helps establish a good cooperative relationship between enterprises and employees, thereby enhancing overall performance.

## 6. Research Conclusions, Theoretical Contributions, Recommendations, and Limitations and Future Directions

### 6.1. Research Conclusions

This study, based on evolutionary game theory, explores the strategy choices and evolutionary patterns of enterprises and employees in HRM value co-creation in the AI era. By constructing a game model and using MATLAB for numerical simulations, we analyzed the impact of factors such as profit distribution, costs, and co-created value distribution on the evolution of strategies between enterprises and employees after the introduction of AI. Specifically, we studied the influence of initial strategy probabilities, enterprise cooperation costs, employee involvement costs, AI profit distribution ratios, and HRM co-created value distribution ratios on equilibrium points and the evolution of strategies.

The research findings indicate that the strategy choices of enterprises and employees exhibit significant mutual influence, which applies not only to AI-driven scenarios, but also to broader technological adoption contexts where cooperation and value co-creation are central. The evolutionary game model shows that the system tends to converge on two equilibrium points: {cooperation, active} and {non-cooperation, passive}, with the former being 2.39 times more likely than the latter. When the initial probabilities of cooperation and involvement are high, both enterprises and employees tend to cooperate and actively become involved, achieving a stable win–win equilibrium state (ESS (1, 1)). Conversely, when initial probabilities are low, the system tends towards a low-efficiency equilibrium state (ESS (0, 0)). Increasing cooperation costs significantly inhibits enterprises’ willingness to cooperate, while higher involvement costs for employees significantly reduce their motivation to become involved. Regarding profit distribution after the introduction of AI, enterprises are more sensitive to profit distribution ratios, and a higher proportion of profit distribution can motivate enterprises to cooperate. Employees, due to their relatively low involvement costs, are inclined to actively become involved as long as there is a certain level of benefit. Moreover, the reasonable distribution of HRM co-created values also significantly affects the cooperation strategies of both parties. A higher proportion of gain distribution can effectively motivate cooperation between enterprises and employees, maximizing the benefits for both. Overall, reducing cooperation costs and establishing reasonable profit-sharing and incentive mechanisms are crucial factors in promoting cooperation between enterprises and employees, to achieve HRM value co-creation.

This study provides theoretical support for HRM strategies of enterprises in the AI era, suggesting that by optimizing cost structures and profit-sharing mechanisms, effective cooperation between enterprises and employees can be promoted, ultimately enhancing overall organizational performance and employee satisfaction.

### 6.2. Theoretical Contributions

This study contributes to evolutionary game theory and the field of Human Resource Management (HRM), particularly in the context of HRM value co-creation with AI involvement, in the following ways:

Firstly, this study expands the theoretical discussion of the impact of emerging technologies like AI on HRM practices. The existing literature predominantly focuses on how AI enhances efficiency and simplifies HRM processes, with relatively limited attention to the dynamic mechanism of AI and HR value co-creation. By constructing an evolutionary game model involving enterprises and employees within technological adoption scenarios, particularly with AI, this study reveals how enterprises and employees jointly select cooperative strategies in an AI environment to achieve HRM value co-creation. It provides a new perspective on understanding the effects of AI on strategy choices, profit distribution, and equilibrium evolution for enterprises and employees.

Secondly, this study contributes to deepening the theory of HRM value co-creation. The concept of value co-creation originated from service-dominant logic, emphasizing value co-creation between enterprises and customers. However, the mechanisms of value co-creation between enterprises and employees in HRM have not been sufficiently explored. This study uses an evolutionary game model to analyze how enterprises and employees jointly create HRM value through strategic choices, and conducts numerical simulations to examine the evolution of strategies under different conditions. The findings show that initial probabilities of cooperation and active involvement, cooperation costs, profit distribution after AI introduction, and co-created value distribution significantly influence the equilibrium of strategy evolution between enterprises and employees. These insights deepen our understanding of how enterprises and employees can jointly realize value co-creation through HRM practices.

Thirdly, this study extends the application of evolutionary game theory to HRM management in an AI context, broadening its application boundaries in management science. Evolutionary game theory is often used to analyze the strategy evolution of individuals or organizations in repeated interactions. This study, by integrating AI’s role in HRM, constructs an evolutionary game model involving enterprises and employees with AI involvement to analyze the impact of AI on strategy choices and equilibrium points. Through model analysis and numerical simulations, the study identifies possible equilibrium states and their stability, and explores the key factors affecting these equilibrium states. This contribution provides a theoretical reference for further application of evolutionary game theory in management practices under technological environments.

Lastly, this study provides new theoretical support for HRM management involving human–AI collaboration. As AI increasingly permeates various aspects of HRM, human–AI collaboration becomes a key means to enhance HRM practice efficiency and value. This study reveals how enterprises can incentivize employees to actively become involved in HRM practices through reasonable profit-sharing mechanisms after AI introduction, and how lowering cooperation costs can increase the propensity of enterprises and employees to cooperate, thereby promoting effective human–AI collaboration. This conclusion offers a theoretical foundation for value co-creation in human–AI collaboration, helping enterprises better achieve human–AI co-creation in practice.

### 6.3. Recommendations

Based on the findings of this study, enterprises and employees should co-create value in HRM with AI involvement by focusing on improving cooperation and involvement willingness, optimizing profit distribution, and building a collaborative environment. The study proposes the following strategic recommendations to effectively promote co-creation between enterprises and employees and enhance the overall performance of HRM practices.

1. Enhance initial cooperation and involvement willingness. To encourage cooperation between enterprises and employees in co-creating value in HRM, enterprises should cultivate a positive organizational culture that fosters cooperation in technological adoption processes, and diversify incentives to enhance employees’ willingness to become involved in HRM. Specifically, enterprises can foster trust between themselves and employees through transparent communication and involved decision-making, thereby increasing the initial probability of cooperation. Simultaneously, employees should enhance their professional skills and knowledge to increase their contribution to HRM, ensuring their irreplaceability, which will further boost their willingness to engage. At the same time, employees should enhance their professional skills and knowledge reserves to increase their contribution to HRM and irreplaceability, thus boosting their willingness to become involved in HRM. This will help both enterprises and employees form a virtuous cycle of cooperation and active involvement, promoting the system toward the stable state of ESS (1, 1).
2. Reduce cooperation costs and enhance cooperation benefits. Reducing enterprise cooperation costs is crucial for maintaining long-term collaboration. Enterprises can adopt AI technologies to optimize HRM processes, streamline workflows, and reduce time and resource investment, thereby effectively lowering cooperation costs and increasing the net benefits of cooperation. Employees should also optimize their working methods to improve efficiency and minimize extra costs in the HRM process. Additionally, enterprises should refine management and resource allocation to balance cooperation costs and benefits, ensuring sustainable collaboration that yields desired outcomes.
3. Reasonably allocate the benefits after AI introduction. The study shows that reasonable benefit allocation plays a key role in motivating cooperation between enterprises and employees. Enterprises should appropriately increase the share of benefits allocated to employees after AI introduction, to encourage active involvement. Enterprises can establish performance-based reward mechanisms, such as bonuses and career development support, to enhance employees’ engagement in HRM activities. Employees should actively become involved in HRM activities involving AI, demonstrate their value, and seek more benefit allocations, thereby enhancing their personal career development opportunities.
4. Optimize the distribution of co-created value in HRM. The reasonable allocation of co-created value in HRM is key to motivating enterprises and employees to jointly become involved in HRM practices. Enterprises should focus on fairness when distributing co-created value, appropriately increasing the share of benefits for employees, thereby encouraging their active involvement in HRM practices. Reasonable profit allocation not only helps to improve employee involvement and satisfaction, but also stimulates employee innovation. Employees should fully understand enterprise goals, actively become involved in co-creating value in HRM, and take advantage of co-created value distribution opportunities to improve their skills and career growth, achieving a win–win situation for both individuals and the enterprise.

### 6.4. Limitations and Future Directions

Although this study has made some theoretical contributions to understanding the co-created value mechanism of HRM between enterprises and employees in the context of AI, several limitations remain. Future research could further improve and expand on these aspects.

First, the evolutionary game model constructed in this study is based on certain assumptions, including that enterprises and employees have bounded rationality and that both costs and benefits can be quantified, which may also apply to other technological adoption contexts beyond AI. However, evolutionary game theory itself has inherent limitations, particularly in modeling complex, dynamic human behaviors and interactions. In the context of AI adoption, there are aspects—such as employee perceptions of AI, ethical concerns, and long-term strategic implications—that are difficult to realistically quantify as payoffs and costs. While these assumptions are necessary for the theoretical model, they may not fully capture the complexities of real-world situations. For instance, the model does not account for factors such as corporate culture, employee psychology, or other psychological factors that may influence decision-making in real-world settings. Future research could incorporate these more realistic aspects of the model and improve its applicability across different organizational contexts.

Second, the numerical simulation method used in this study validates the cooperation and co-creation process between enterprises and employees under AI through the evolution of strategic paths with different parameter settings. However, since the parameters used in the numerical simulation are mainly based on expert opinions and empirical estimates, this may introduce a limitation in terms of model precision. These parameters may not fully reflect the behavioral characteristics of enterprises and employees across diverse organizational contexts and industries. Therefore, future research could employ field studies, case studies, or experimental data to further verify the predictive power of the model, ensuring the representativeness and reliability of the results. 

In addition, this study does not delve into how the specific technical features of AI in HRM impact strategic choices by enterprises and employees. The diversity of AI technologies may have different impacts on the cooperative mechanisms within HRM practices, thereby influencing the process of co-created value between enterprises and employees. Future research could analyze the differential effects of various AI technologies, such as machine learning, natural language processing, and predictive analytics, on strategy evolution and cooperation. Incorporating specific AI application scenarios into the research would provide more precise and practical guidance for enterprises.

Lastly, another limitation of this study lies in its insufficient consideration of the dynamic changes in external environmental factors, such as policy changes, market competition, and economic fluctuations, and their impact on the strategic choices of enterprises and employees. These dynamic factors—such as changes in legislation, market shifts, and economic crises—could significantly influence the evolution of both parties’ strategies. Furthermore, the introduction of competition among multiple firms and employees could provide a more comprehensive understanding of how strategic decisions evolve under external pressures. Therefore, future research could introduce dynamic factors from the external environment to improve the model’s complexity, thereby providing a better explanation of enterprise and employee behavior in different macro environments.

In light of these limitations, future research can further refine the study by addressing more realistic model assumptions, incorporating empirical data, exploring differences in AI technological characteristics, and considering dynamic external environments to deepen the understanding of the co-created value mechanism of HRM between enterprises and employees under AI. This will contribute to providing enterprises with more systematic and comprehensive theoretical support for effectively practicing HRM in the AI era and achieving joint development for enterprises and employees.

## Figures and Tables

**Table 1:** Payoff matrix of enterprises and employees with AI involvement.

Enterprises	Employees	
	Active (q)	Passive (1 − q)
Cooperation (p)	$\mathrm{Enterprises}:\;R_{2}$ $+\;a\Delta \mathrm{AI}+b\Delta R_{h}$ $-(C_{2}$ $+\;\Delta C_{AI}$)	$\mathrm{Enterprises}:\;R_{2}$ $+\;k\Delta \mathrm{AI}-(C_{2}$ $+\Delta C_{AI}$)
	${Employees:r}_{2}$ $+\;(1-a)\Delta \mathrm{AI}+(1-b)\;\Delta R_{h}$ $-(c_{2}$ $-\Delta c_{AI}$)	$Employees:r_{1}\prime$ $-\;c_{1}\prime$
Non-cooperation (1 − p)	$\mathrm{Enterprises}:\;R_{1}$ $\;+\;\Delta R-C_{1}$	$\mathrm{Enterprises}:\;R_{1}$ $\;-\;C_{1}$
	$Employees:r_{2}$ $\;-\;c_{2}$	Employees: $r_{1}$ $\;-\;c_{1}$

**Table 2:** Numerical expressions of equilibrium points.

Equilibrium Point	det(J) Expression	tr(J) Expression
**A**(0, 0)	$D_{1}d_{1}$	$D_{1}+d_{1}$
**B**(0, 1)	$-(D_{1}+d_{1})\;d_{1}$	$D_{1}+D_{2}-d_{1}$
**C**(1, 0)	$-D_{1}$$(d_{1}+d_{2}$)	$-D_{1}+d_{1}+d_{2}$
**D**(1, 1)	$(D_{1}+d_{1})\;$$(d_{1}+d_{2}$)	$-(D_{1}+D_{2}+d_{1}+d_{2})$
$E(p_{0}$$,\;q_{0}$)	$-[p_{0}(1-p_{0})D_{2}]$$[q_{0}(1-q_{0})d_{2}$]	0

$D_{1}$, $D_{2}$: Parameters related to enterprises, $d_{1}$, $d_{2}$: Parameters related to employees.

**Table 3:** Evolutionary stable points for enterprises and employees.

Equilibrium Point	Symbol of det(J)	Symbol of tr(J)	Stability Result
**A**(0, 0)	+	−	ESS
**B**(0, 1)	+	+	Unstable
**C**(1, 0)	+	+	Unstable
**D**(1, 1)	+	−	ESS
$E(p_{0}$$,\;q_{0}$)	−	0	Saddle Point

**Table 4:** Parameter setting of the evolutionary game model of enterprises and employees under AI involvement.

Parameter Classification	Symbol	Parameter Description	Value
Enterprises’ Costs and Benefits	$R_{1}$	Enterprises’ benefits when “non-cooperative”	80
	$R_{2}$	Enterprises’ benefits when “cooperative”	120
	ΔR	Incremental benefit from non-cooperation but active employee involvement	5
	$\Delta R_{h}$	Co-created value from enterprise–employee cooperation	40
	ΔAI	Additional benefit from AI introduction	40
	$C_{1}$	Cost of non-cooperation	30
	$C_{2}$	Cost of cooperation	70
	$\Delta C_{AI}$	Cost of AI introduction	20
Employees’ Costs and Benefits	$r_{1}$	Employees’ benefits during passive involvement	40
	$r_{1}$′	Employees’ short-term benefit when passive but enterprise cooperates	35
	$r_{2}$	Employees’ benefit during active involvement	45
	$\Delta r_{AI}$	Additional benefit from AI introduction for employees	10
	$c_{1}$	Employees’ cost during passive involvement	20
	$c_{1}$′	Cost for employees who are passive but enterprises cooperate	25
	$c_{2}$	Cost for active involvement	40
	$\Delta c_{AI}$	Cost reduction due to AI introduction	5
Benefit Distribution Ratios	a	Enterprises’ share of benefits from AI introduction	0.6
	1 − a	Employees’ share of benefits from AI introduction	0.4
Employees’ Costs and Benefits entry 3	b	Enterprises’ share of cooperative co-created values	0.5
	1 − b	Employees’ share of cooperative co-created values	0.5
	k	Enterprises’ share of automated benefits with passive employee involvement	0.3

## Data Availability

The datasets generated during and/or analyzed during the current study are available from the corresponding author upon reasonable request.
